# Corrosion Properties of Mn-Based Alloys Obtained by Aluminothermic Reduction of Deep-Sea Nodules

**DOI:** 10.3390/ma14185211

**Published:** 2021-09-10

**Authors:** Šárka Msallamová, Pavel Novák, Pauline Miossec, Jaromír Kopeček, Alisa Tsepeleva, Darya Rudomilova, Jaroslav Fojt

**Affiliations:** 1 Department of Metals and Corrosion Engineering, University of Chemistry and Technology, Prague, Technická 5, 166 28 Prague 6, Czech Republic; msallams@vscht.cz (Š.M.); pauline.miossec@etu.unilim.fr (P.M.); tsepelel@vscht.cz (A.T.); fojtj@vscht.cz (J.F.); 2 FZU-Institute of Physics of the Czech Academy of Sciences, Na Slovance 2, 182 00 Praha 8, Czech Republic; kopecek@fzu.cz; 3 Technopark Kralupy, University of Chemistry and Technology, Prague, G. Karse 7/2, 278 01 Kralupy nad Vltavou, Czech Republic; darya.rudomilova@vscht.cz

**Keywords:** deep-sea nodules, electrochemical measurement, aluminothermic reduction, Mn–Al alloy, intergranular corrosion

## Abstract

Deep-sea manganese nodules are polymetallic oxidic ores that can be found on a seabed. Aluminothermic reduction is one of the possibilities of manganese nodules processing. This process obtains the polymetallic alloy with a high content of Mn and a varying content of Al, depending on the ratio between aluminum and nodules. The corrosion behaviors of three experimental Mn-based alloys produced by aluminothermic reduction with a content of Mn > 50 wt % were studied. The electrochemical testing in potable water and model seawater was used to explain the corrosion mechanism of Mn-based alloys. The results showed that the corrosion rate of experimental Mn-based alloy decreases with the increase in aluminum content in both potable water and model seawater. It was observed that the uniform corrosion of experimental Mn-based alloys is changed with an increase in aluminum content in alloy to localized corrosion, which was caused by microcells in an environment of model seawater. In contrast, the formation of a semi-protective layer of corrosion products was observed on the surface of Mn-based alloys with a higher content of aluminum in potable water. Moreover, the pitting corrosion of tested Mn-based alloys was observed neither in potable water nor in model seawater.

## 1. Introduction

Deep-sea nodules are polymetallic oxidic ores, which can be found on a seabed. One of the main deposits of the deep-sea nodules is Clarion–Clipperton Fracture Zone in the Pacific Ocean. There are about 100 billion tons of deep-sea nodules at a depth of 4000–6000 m [1,2,3,4]. Deep-sea nodules have high porosity and a large specific surface. Their size ranges from a few millimeters to 30 cm [5,6]. There are several types of deep-sea nodules. The main types include manganese nodules, sedimentary nodules, and nodules formed by volcanic activity [1]. Economically, the most interesting ones are the deep-sea manganese nodules. The manganese nodules are polymetallic ores that also contain other metals, mainly Fe, Ni, Co, and Cu. They can also contain Zn, Mo, Ba, Mg, Ti, V, etc. as minority metallic impurities [1,3]. The major crystalline phases of manganese nodules are Todorokite (mixed oxides of Mn, Mg, Ca, Na, and K), Buserite (mixed hydrated oxides of Mn and Na), Birnessite (mixed hydrated oxides of Mn, Ca, and Na), and Vernadite (δ-MnO_2_) [6,7,8]. The rest of the manganese nodules contain clay, quartzite or zeolite, and amorphous hydrated ferric oxide [9,10].

Manganese, which is the main constituent of the above-mentioned deep-sea nodules, is used in metallurgy mainly as the alloying element. The highest portion of manganese produced worldwide is used as an alloying element in steelmaking. The role of manganese in steel is the de-oxidation and strengthening of the solid solution and improvement of the heat treatment response [11]. Manganese also improves the corrosion behavior of the steel, thanks to the adsorption of Mn^2+^ ions on the steel surface, inhibiting the corrosion and forming manganese compounds on the surface [12].

The Al–Mn alloys with a content of Mn up to 1.5 wt % are often commercially used in automotive, chemical, and construction industries. The Al–Mn alloys cannot be strengthened by heat treatment, and their ductility decreases with the increase in manganese in these alloys [13,14]. In contrast, the Al–Mn alloys have good corrosion resistance in atmospheric or soil conditions and seaside environments [13].

The results of some studies describe a better corrosion resistance of Al–Mn alloys with a content of Fe of 0.4–0.8 wt %, compared with a commercial Al (with a Fe content < 0.4 wt %) in atmospheric conditions [15]. The higher atmospheric-corrosion resistance of Al–Mn alloys with some content of Fe is caused by smaller potential differences between the solid solution of Al, enriched by Mn, and intermetallic phase Fe_3_Al enriched by Mn compared with commercial Al [15]. However, the corrosion rate of a pure Al in atmospheric conditions is comparable to the corrosion rate of Al–Mn alloys in the same environment [13,16].

The corrosion resistance of Al–Mn alloys caused by the pitting corrosion is higher compared to pure Al, and it increases with the increase in manganese content in these alloys [13]. The main reason is the formation of a more compact passive layer on the surface of Al–Mn alloys. Al_2_O_3_ mostly forms the passive layer of Al-Mn alloys [17,18]. The selective dissolution of manganese from the metal matrix improves the adherence of the passive layer to a surface of an Al–Mn alloy, and it leads to the decrease in internal stress in the passive layer [17,18]. The number of defects in the passive layer decreases with the increase in manganese in Al–Mn alloys. This effect also leads to the increase in the protective character of the Al–Mn alloys’ passive layer [19,20]. The content of manganese in Al–Mn alloys also changes the kinetics of pits formation in Al–Mn alloys. The results of some studies present that a higher current density is necessary for the formation of pitting corrosion in Al–Mn alloys when compared to a pure Al [20]. In contrast, the Al–Mn alloys have a worse resistance to intergranular corrosion compared to pure Al. The formation of depleted zones along the grain boundaries of a solid solution of Al in the places where the cathodic intermetallic phases are formed leads to intergranular corrosion of Al–Mn alloys [13,16]. The aluminum alloy with a higher content of manganese (14 wt %) was the subject of the experiments carried out by D. Shechtman [21], where the quasicrystalline phase had been discovered.

Over the decades, functional materials with a high content of manganese have been developed, such as Hadfield steel or Heusler alloys. Hadfield steel is a ferrous material with a high manganese content (approx. 13 wt %) [22,23]. It stabilizes the austenitic structure of the steel at room temperature. When such steel is loaded, the austenite transforms into martensite, which strengthens the material and makes it more wear resistant. The Heusler alloys are the phases of A_2_BC stoichiometry, which can exhibit ferromagnetic behavior, even if they do not contain any ferromagnetic element. Many of these phases, such as Cu_2_MnAl [24] or recently described Mn_2_FeSi and Mn_2_FeAl phases [25,26], are the manganese-containing ones.

From the above overview, there are high-manganese materials that exhibit very interesting properties, which can bring useful applications. Therefore, we focused on manganese-based materials in this study, which can be prepared simply from the deep-sea nodules. For the processing method, we selected the direct aluminothermic reduction, which was recently proved as a feasible technology [8]. The reasons for choosing this method without any separation of the present elements are the following:

- The deep-sea nodules are potential sources of metals after the reserves in the soil are consumed.
- The direct aluminothermic reduction of the polymetallic ore is an eco-friendly way of how to get the metallic material. It requires much lower energy than the hydrometallurgical and pyrometallurgical processes proposed recently for the processing of deep-sea nodules, and it generates much less emissions.
- The obtained polymetallic alloy with a natural ratio of the alloying elements can bring new functional properties.

Recently we described the microstructure, phase composition, and mechanical and tribological properties of the materials obtained by the aluminothermic reduction with various amounts of aluminum. It was revealed that these materials exhibit high hardness and very good wear resistance. The hardness reaches 732, 790, and 813 HV1 for alloy reduced using stoichiometric amount and 10% and 20% excess of aluminum, respectively. The wear resistance was found to be comparable with that of the AISI D2 tool steel, but without the requirement of any heat treatment [8]. However, the materials are relatively brittle [8]. Therefore, the alloys can be potentially used for the wear resistant coating rather than the bulk materials. Due to the presence of Heusler phases in the alloys prepared using aluminothermy with a high amount of aluminum, these materials are expected to be ferromagnetic, which can further extend their potential application range. However, the corrosion behavior of these alloys was not known until now. Therefore, the aim of the study is to describe the corrosion behavior of polymetallic Mn-based alloys prepared by the aluminothermic reduction of deep-sea nodules in both potable water and model seawater.

## 2. Materials and Methods

Experimental Mn-based alloys used for corrosion testing were prepared by aluminothermic reduction of deep-sea nodules, according to the general Equation (1).

3Me_x_O_y_ + 2yAl → 3xMe + yAl_2_O_3_ (1)

The average elemental composition of deep-sea nodules is presented in Table 1.

Three types of Mn-based alloys were prepared by aluminothermic reduction:

- with a stoichiometric content of aluminum (0% Al),
- with 10% surplus of aluminum (10% Al) and
- with 20% surplus of aluminum (20% Al).

The alloys were milled, and spark plasma sintered at 1000 °C for 10 min, using the pressure of 48 MPa and the heating rate of 300 °C/min. The elemental composition of prepared Mn-based alloys is presented in Table 2. The rest of the deep-sea nodules’ elements—Mg, Ca, Na, Zn, and Ti (Table 1)—became part of the slag, which was formed during the aluminothermic reduction.

The elemental composition of deep-sea nodules and the experimental Mn-based alloys were determined by the X-ray fluorescence (XRF) spectrometer ARL 9400 XP (Thermo ARL, Ecublens, Switzerland).

The microstructure of experimental alloys was observed by using the metallographic optical microscope NIKON Eclipse MA200 (Nikon, Tokyo, Japan), after etching by 10% nital (10 % solution of HNO_3_ (PENTA, Prague, Czech Republic) in ethanol).

The Gamry PCI4 instrument (Gamry Instruments Inc., Warminster, PA, USA) was used for the testing of corrosion behavior of the experimental alloys. The measurement was performed in the solution of potable water (pH = 5.7) and model seawater containing 3.5 wt % Cl l^−1^ at room temperature (pH = 6.5). The obtained data were evaluated using Gamry Framework software (Gamry Instruments Inc., Warminster, PA, USA). A saturated calomel (Hg_2_Cl_2_) electrode (SCE) was used as a reference electrode, while platinum wire was applied as a counter electrode. The polarization resistance (Rp) and potentiodynamic anodic curves (PDA) and potenciodynamic cathodic curves (PDC) were measured. The potentiostatic polarization at E = −0.145 V/SCE for 20 min was used to explain the corrosion mechanism of Mn-based alloys in the model seawater. The polarization resistance (Rp) was measured according to Equation (2):

(2) $$R_{P}={\left(\frac{\Delta E}{\Delta i}\right)}_{\Delta E\to 0\;}$$

where E is an electrochemical potential and i is the current.

The polarization resistance value is used for calculating the corrosion current density (i_corr_) by using Stern–Geary Equations (3) and (4):

(3) $$i_{\mathrm{corr}}=\frac{B}{R_{P}}$$

(4) $$B=\frac{b_{a}\cdot b_{c}}{2\cdot 3\cdot \left(b_{a}+b_{c}\right)}$$

where b_a_ and b_c_ represent the anodic and cathodic Tafel slope, respectively [27].
Parameters of the Rp measurement:time of OCP equilibration, 1800 spolarization range ± 0.02 V/SCEscan rate 0.125 mVs^−1^Parameters of the PDA measurement: time of equilibration of OCP, 1800 spolarization range from −0.02 V/OCP to 1 V/SCEscan rate 1 mVs^−1^Parameters of the PDC measurement:time of equilibration of OCP, 1800 spolarization range from 0.02 V/OCP to −1.2 V/SCEalic>12) scan rate 1 mVs^−1^

The concentration of cations in solution were analyzed by atomic absorption spectrometer (AAS) Agilent 280 FS AA (Agilent Technologies, Mulgrave, Australia).

The phase composition of corrosion products was determined by DXR^TM^3 (DXR) Raman Microscope (ThermoFisher Scientific, Waltham, MA, USA).

The surface potential differences of Mn–Al alloys were mapped with SmartSPM 1000 (AIST-NT Inc., Novato, CA, USA) Atomic Force Microscope (AFM).

The composition of the passive layer of Mn-based alloys was determined by X-Ray Photoelectron Spectroscopy (XPS), ESCAProbe (Omicron Nanotechnology, Taunusstein—Neuhof, Germany).

## 3. Results and Discussion

**Figure 1:**
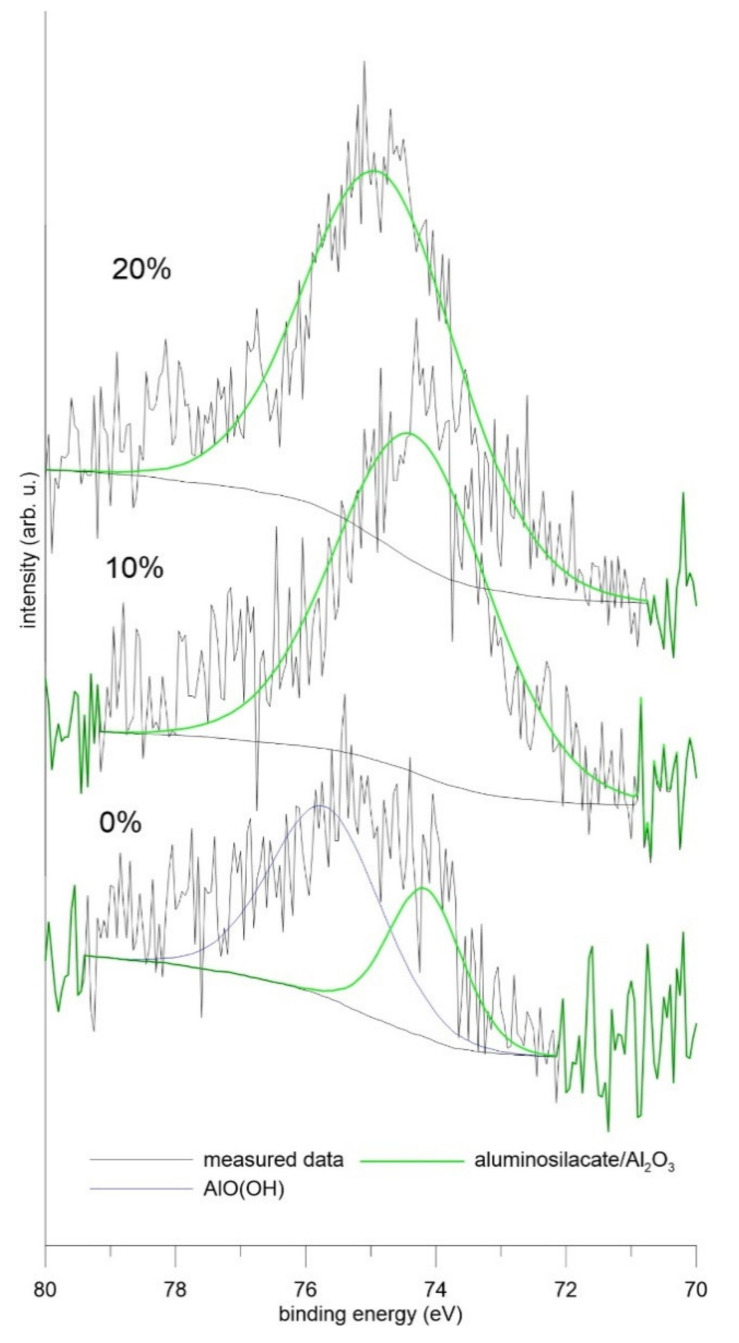
Detail of the XPS spectra of tested alloys—the original surface before the exposure.

At first, the analysis of the natural layers of corrosion products on the ground samples of all tested alloys was carried out by means of the X-Ray Photoelectron Spectroscopy. The results showed that the layer contained higher levels of aluminum and silicon (see Table 3). By the detailed analysis of the XPS spectra, it was found that the natural layer of corrosion products formed predominantly by the aluminosilicate in the case of all alloys, while in the case of the alloy reduced in stoichiometric amount, there was also the AlO(OH) phase as the constituent of the layer (Figure 1). In the case of manganese, which was also present in the surface layer, there were overlaps between individual oxides, so it was impossible to determine a specific oxidation state from the XPS results.

The results of the electrochemical tests showed that the increase in aluminum content in the Mn-based alloys led to an increase in corrosion resistance in both potable water and model seawater. The corrosion resistance of Mn-based alloys in potable water was higher compared to model seawater (Table 4). The formation of a semi-protective layer of corrosion products on the surface of Mn-based alloys with a higher content of aluminum (10% Al, 20% Al) together with low content of chlorides were the reasons for a lower corrosion rate of Mn-based alloys in potable water.

**Figure 2:**
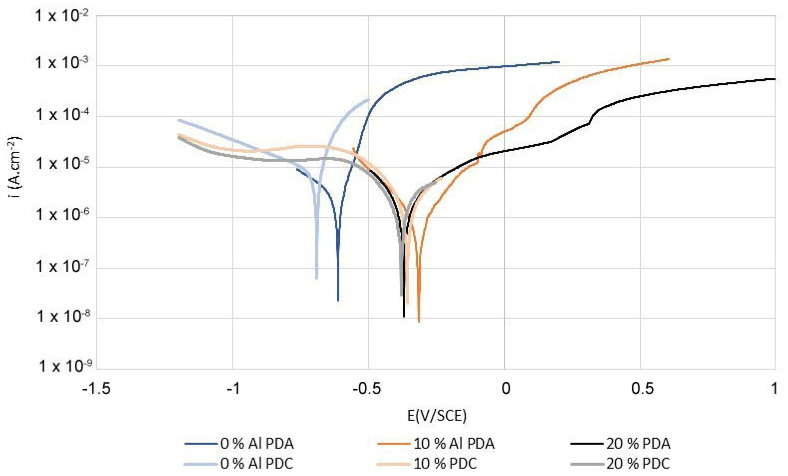
Comparison of PDA curves in potable water.

The range of semi-protective layer of corrosion products of Mn-based alloy with 10% Al surplus in potable water was from approximately −0.06 V/SCE to 0.08 V/SCE, and the current density was approximately 4.5 × 10^−5^ A·cm^−2^ in this range (Figure 2). The X-Ray Photoelectron Spectroscopy measurement showed that the semi-protective layer contained higher levels of aluminum and silicon, also after the exposure (Table 3), indicating the formation of aluminosilicate. Manganese was also present in the passive layer in ratio Al:Mn of approximately 17:1 (Table 3). The range of semi-protective layer of corrosion products of Mn-based alloy with 20% Al surplus in potable water was wider, approximately from −0.14 V/SCE to 0.31 V/SCE, and the current density was lower, approximately 2.6 × 10^−5^ A·cm^−2^ in this range, comparing to Mn-based alloy with 10% Al surplus (Figure 2). The X-Ray Photoelectron Spectroscopy measurement (XPS) showed that the semi-protective layer also formed by aluminosilicate with the admixture of Mn in atomic ratio Al:Mn = 28:1 (Table 3). The quality of the semi-protective layer of corrosion products was influenced by the presence of a cathodic Mn_2_Fe(Si,Al) intermetallic phase, significantly enriched by Cu and Ni (see below, together with Mn_2_P [8]. These cathodic intermediary phases influenced the range of semi-protective layer of corrosion products of Mn-based alloys with 10% and 20% Al surplus. The extent of the “plateau” increased with the growing amount of aluminum in the matrix (Figure 2). The cathodic reaction was controlled by oxygen diffusion in potable water in all cases (Figure 2).

**Figure 3:**
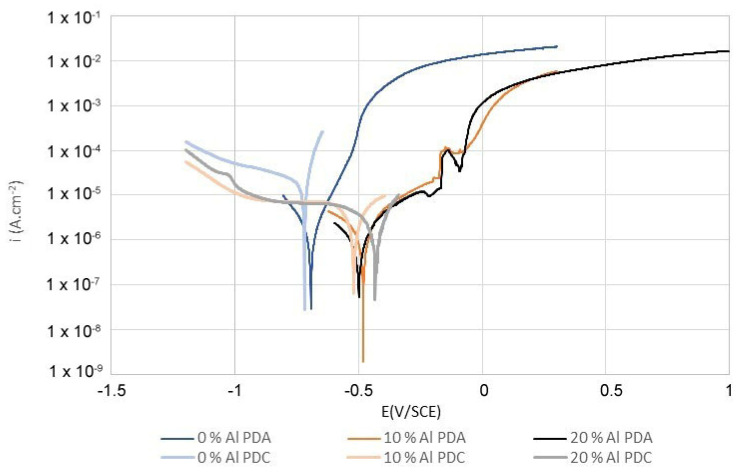
Comparison of PDA curves in model seawater.

As opposed to that, the formation of a semi-protective layer of corrosion products on a surface of Mn-based alloys with 10% and 20% surplus of aluminum (10%Al and 20%Al) was not observed in model seawater (Figure 2). On the contrary, a significant localized corrosion attack was observed at −0.145 V/SCE for both Mn-based alloys (Figure 3).

The cathodic reaction was controlled by oxygen diffusion in seawater in all cases (Figure 3).

The formation of a semi-protective layer of corrosion products on the surface of Mn-based alloy with a stoichiometric amount of aluminum was not observed in potable water nor model seawater (Figure 2 and Figure 3). The matrix phase, identified recently as β-Mn_66_Ni_20_Si_14_ [8], corroded uniformly mainly according to Equations (5) and (6):

(5) $$\mathrm{Mn}\to {\mathrm{Mn}}^{2+}+2e^{-}$$

(6) $$H_{2}O+\frac{1}{2}O_{2}+2e^{-}\to 2{\mathrm{OH}}^{-}$$

The pure metals Fe, Cu, Al, Mn, and Ni, which were contained in Mn-based alloys (Table 2), were also electrochemically tested in both potable water and model seawater as a reference. The effect of Co on the corrosion behavior of Mn-based alloys was neglected. The results showed that the difference between the value of open circuit potential (OCP) of Mn and open circuit potential of Cu and Ni was approximately 1.2 V in both potable water and model seawater (Table 5). The potential gradient between the above-mentioned elements was the reason for localized corrosion damage of Mn-based alloy with a higher content of aluminum, which was observed in the model seawater (Figure 3).

### Localized Corrosion of Mn-Based Alloys with Higher Content of Aluminium in Model Seawater

Mn-based alloys with 10% and 20% surplus of aluminum were significantly damaged by corrosion attack in model seawater at −0.145 V/SCE, as was mentioned above (Figure 3). The electrochemical testing by potentiostatic polarization at E = −0.145 V/SCE of all three Mn-based alloys in model seawater was used to explain this corrosion mechanism. Model seawater was analyzed by AAS after potentiostatic polarization. The measured concentration of cations was used for the calculation of the corrosion rate of elements in the material (Table 6). The corrosion rate of elements was evaluated vs. the chemical composition of intermediary phases and the metal matrix of Mn-based alloys. The mapping of the surface potential differences of Mn-based alloys completed these results.

**Figure 4:**
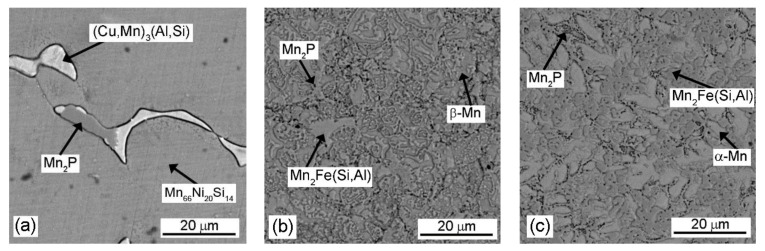
Optical micrographs of Mn-based alloys: (**a**) 0% Al, (**b**) 10% Al, and (**c**) 20% Al.

The alloys are composed of a manganese-rich matrix, the intermetallic phase, and Mn_2_P (Figure 4) [8]. The crystal structure of the matrix changes from β-Mn_66_Ni_20_Si_14_ solid solution with P2_1_3 symmetry for “0% Al”, through β-Mn (P4_1_32 structure) for “10% Al” to α-Mn (I-43m) in “20% Al” alloy, as described in [8]. The intermetallic phase is (Cu,Mn)_3_(Al,Si), with the Pm-3m structure in the alloy reduced using the stoichiometric amount of aluminum (Figure 4a). As the content of aluminum in the alloy increased, this phase was replaced by the Mn_2_Fe(Si,Al) Heusler phase [8], see Figure 4b,c. In the previous work [8], other minor phases, such as manganese silicide, have been detected in the alloys after aluminothermic reduction with 10% and 20% surplus of aluminum. However, these phases were not recognized in the sintered materials studied in this work. SPS sintering probably led to the homogenization of the sample.

Both the intermetallic phase and the Mn-based solid solution were enriched by elements Fe, Ni, and Cu (Table 7). It needs to be emphasized that the intermetallic phase contained 2 to 20 times more Cu and Ni than the solid solution based on Mn in all experimental Mn-based alloys (Table 7).

**Figure 5:**
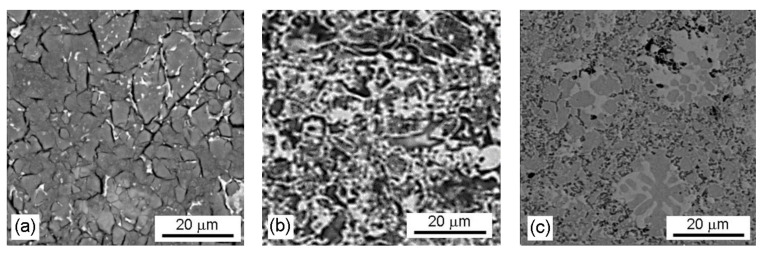
SEM micrographs (BSE) of the exposed surfaces of Mn-based alloys after the corrosion test in model seawater: (**a**) 0% Al, (**b**) 10% Al, and (**c**) 20% Al.

The localized corrosion attack, which was observed in the Mn-based alloy with 10% and 20% surplus of aluminum in model seawater at E = −0.145 V/SCE, was caused by microcells. The cathodic places were the intermediary phase Mn_2_Fe(Si,Al) together with Mn_2_P, significantly enriched by elements Cu and Ni, compared with the anodic place—the Mn-based solid solution (Table 7). The corrosion caused by microcells led to the preferential anodic dissolution of the solid solution (Figure 5b,c—dark places without corrosion products) along the phase interface of intermetallic phase Mn_2_Fe(Si,Al) (Figure 5b,c—bright places), and it led to the localized corrosion attack of both Mn-based alloys (Figure 2, Table 6).

The localized corrosion attack caused by microcells was not observed in the Mn-based alloy with the stoichiometric ratio (0% Al) of aluminum at E = −0.145 V/SCE (Figure 3). This Mn-based alloy corroded uniformly probably according to Equations (5)–(8) [28]:

(7) $$2{\mathrm{Mn}}^{2+}+3H_{2}O\to {\mathrm{Mn}}_{2}O_{3}+6H^{+}+2e^{-}$$

(8) $${\mathrm{Mn}}_{2}O_{3}+H_{2}O\to 2{\mathrm{MnO}}_{2}+2H^{+}+2e^{-}$$

**Figure 6:**
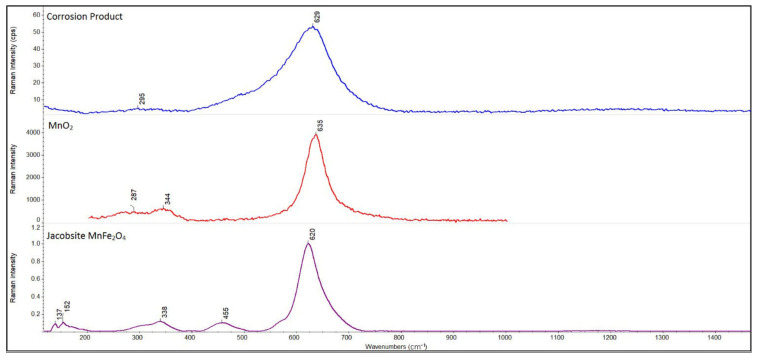
Raman’s spectrum of the Mn-based alloy corrosion product with the stoichiometric ratio of aluminum (exposed in model seawater).

The uniform layer of corrosion products composed of amorphous mixed oxide based on MnO_2_, containing a small content of amorphous mixed iron oxides (determined by DXR), covered the surface [29] (Figure 5a and Figure 6) after potentiostatic polarization at E = −0.145 V/SCE.

The intermetallic phase (Cu,Mn)_3_(Al,Si) was also visible in the layer of corrosion products (Figure 5a—bright places).

**Figure 7:**
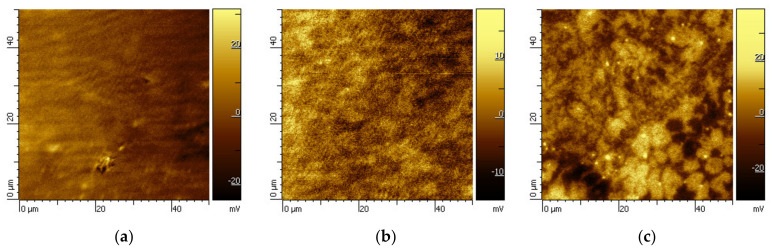
AFM-SKP maps of the surface of the polished Mn-based alloys: (**a**) 0% Al, (**b**) 10% Al, and (**c**) 20% Al.

The results mentioned above correspond to the mapping of the surface potential differences. Significant discontinuities in values of potentials were not visible on the surface of the Mn-based alloy with a stoichiometric content of aluminum (0% Al) (Figure 7a). Conversely, with the increase in aluminum content in Mn-based alloys, the increase in the number of localized places with a higher potential difference in a metal matrix (10% Al, 20% Al) was observed. The places with a higher potential difference correspond to the intermediary phase Mn_2_Fe(Si,Al) and Mn_2_P (Figure 7b,c—the bright places). The mutual potential difference between intermediary phases Mn_2_Fe(Si,Al) and Mn_2_P and the β-Mn solid solution was up to 60 mV (Figure 7b,c).

## 4. Conclusions

The key findings of the presented research can be summarized as follows:

- The corrosion rate of Mn-based alloys obtained by the aluminothermic reduction of deep-sea nodules was higher in model seawater compared to potable water.
- The corrosion rate of the tested alloys decreased with the increase in aluminum content in both potable water and model seawater.
- The formation of a semi-protective layer of corrosion products was observed only on the surface of Mn-based alloys with higher content of aluminum in potable water. The semi-protective layer of corrosion products was composed of aluminosilicate with the admixture of Mn. The atomic ratio of Al:Mn in a semi-protective layer of corrosion products was increasing in favor of aluminum with the increase in aluminum content in Mn-based alloy.
- The Mn-based alloys with a higher content of aluminum (the aluminum surplus of 10 and 20% during aluminothermic reduction) were damaged by a significant localized corrosion attack in model seawater. The mentioned localized corrosion attack was caused by galvanic microcells. The intermediary phase Mn_2_Fe(Si,Al) together with Mn_2_P, significantly enriched by Cu and Ni, were the cathodic places in the anodic β-Mn solid solution. The mutual potential difference between intermediary phases and a solid solution of β Mn was up to 60 mV. On the other hand, the Mn-based alloy reduced using the stoichiometric amount of aluminum corroded uniformly, and the effect of corrosion caused by microcells was not observed in the model seawater. The reason is probably the presence of a different intermediary phase (Cu,Mn)_3_(Al,Si) and its low volume fraction.
- From the above findings and the previously published results about the wear resistance, it can be concluded that the alloys with lower amounts of aluminum are more corrosion-resistant, while the materials with higher aluminum content are characterized by higher wear resistance. Their future application can be especially in the form of protective coatings, which can resist well in potable water, are wear resistant, and probably have negligible toxicity. The use in salt water is possible in the case of the low-aluminum one (with 0% surplus of aluminum).

## Figures and Tables

**Table 1:** The average composition of deep-sea nodules (XRF).

Element	Mn	Fe	Si	Al	Mg	Ca	Na	Cu	Ni	Zn	Ti	Co
(wt %)	30.6	4.4	3.5	2.2	1.9	1.8	1.6	1.2	1.1	0.2	0.4	0.1

**Table 2:** The average composition of experimental Mn-based alloys (XRF).

Element (wt %)	Mn	Fe	Si	Al	Cu	Ni	Co	P
0 % Al	bal.	20.7	8.5	0.8	4.5	6.4	0.5	0.5
10 % Al	bal.	19.6	8.3	5.6	5.9	6.7	0.6	0.3
20 % Al	bal.	15.4	8.0	9.3	3.9	4.1	0.5	0.5

**Table 3:** Chemical composition of the oxide layer on tested alloys (XPS, at. %) before the exposure (ground surface) and after the exposure to −0.145 V/SCE.

Alloy	Exposure	S	Si	Al	Mn	Fe	Cu	Zn	O
0% Al	-	-	21	11.9	6.3	2.7	0.8	1.4	bal.
	potable water	-	22.4	3.7	3	2.7	0.3	0.4	bal.
	seawater	-	19	6.2	4.5	3	0.6	1.2	bal.
10% Al	-	-	21	16.7	3.4	2.4	0.5	0.6	bal.
	potable water	5.8	24.1	14.9	0.9	0.7	0.5	-	bal.
	seawater	-	31	17.7	0.8	0.4	0.8	-	bal.
20% Al	-	4.8	15.9	19.9	1.4	0.9	0.8	-	bal.
	potable water	5.1	5.1	30.9	1.1	0.5	0.4	-	bal.
	seawater	4.8	10.5	24.1	0.8	0.4	1.2	-	bal.

**Table 4:** Comparison of Mn–Al alloys corrosion properties.

Alloy	Potable Water		Seawater	
	Rp(Ω·m^2^)	OCP(V/SCE)	Rp(Ω·m^2^)	OCP(V/SCE)
0% Al	0.416	−0.610	0.470	−0.651
10% Al	2.138	−0.357	1.572	−0.513
20% Al	2.701	−0.336	1.230	−0.452

**Table 5:** Comparison of the pure metals’ corrosion properties.

	Potable Water		Seawater	
Element	Rp(Ω·m^2^)	OCP(V/SCE)	Rp(Ω·m^2^)	OCP(V/SCE)
Fe	0.365	−0.748	0.163	−0.719
Cu	1.551	−0.127	0.816	−0.204
Al	0.559	−0.516	0.493	−0.726
Mn	0.332	−1.366	0.259	−1.343
Ni	14.51	−0.087	15.37	−0.138

**Table 6:** The corrosion rate of elements in model seawater at E = −0.145 V/SCE.

v_corr_ (g m^−2^ h^−1^)	Cu	Mn	Fe	Al	Ni
0% Al	<0.1	16.1	4.3	0.9	<0.1
10% Al	<0.1	1.9	0.8	<0.5	<0.1
20% Al	<0.1	1.5	<0.5	1.2	<0.1

**Table 7:** Elemental composition of intermediary phases and the matrix formed by the solid solution (SEM/EDS).

Element (wt %)		Mn	Fe	Al	Ni	Cu	Si
0% Al	intermetallics	37.6	5.0	9.5	4.0	41.4	2.5
	solid solution	60.1	18.4	1.7	3.8	2.0	14.0
10% Al	intermetallics	46.3	14.5	18.3	5.3	6.9	8.7
	solid solution	58.5	15.6	6.1	3.3	2.5	14.0
20% Al	intermetallics	41.6	16.6	25.1	5.8	5.5	5.4
	solid solution	61.8	14.2	6.8	1.8	1.4	14.0
